# Open-source curation of a pancreatic ductal adenocarcinoma gene expression analysis platform (pdacR) supports a two-subtype model

**DOI:** 10.1038/s42003-023-04461-6

**Published:** 2023-02-10

**Authors:** Luke A. Torre-Healy, Ryan R. Kawalerski, Ki Oh, Lucie Chrastecka, Xianlu L. Peng, Andrew J. Aguirre, Naim U. Rashid, Jen Jen Yeh, Richard A. Moffitt

**Affiliations:** 1 Department of Biomedical Informatics, Stony Brook Medicine, Stony Brook, NY USA; 2 Department of Pathology, Stony Brook Medicine, Stony Brook, NY USA; 3 Department of Pharmacological Sciences, Stony Brook Medicine, Stony Brook, NY USA; 4 Department of Pharmacology, University of North Carolina, Chapel Hill, NC USA; 5 Lineberger Comprehensive Cancer Center, University of North Carolina, Chapel Hill, NC USA; 6 Department of Medical Oncology, Dana-Farber Cancer Institute, Boston, MA USA; 7 Department of Biostatistics, University of North Carolina at Chapel Hill, Chapel Hill, NC USA; 8 Department of Surgery, University of North Carolina, Chapel Hill, NC USA; 9 Department of Biomedical Informatics, Emory University, Atlanta, GA USA; 10 Department of Hematology and Medical Oncology, Emory University, Atlanta, GA USA

**Keywords:** Pancreatic cancer, Software, Gene expression, Pancreatic cancer, Quality control

## Abstract

Pancreatic ductal adenocarcinoma (PDAC) is an aggressive disease for which potent therapies have limited efficacy. Several studies have described the transcriptomic landscape of PDAC tumors to provide insight into potentially actionable gene expression signatures to improve patient outcomes. Despite centralization efforts from multiple organizations and increased transparency requirements from funding agencies and publishers, analysis of public PDAC data remains difficult. Bioinformatic pitfalls litter public transcriptomic data, such as subtle inclusion of low-purity and non-adenocarcinoma cases. These pitfalls can introduce non-specificity to gene signatures without appropriate data curation, which can negatively impact findings. To reduce barriers to analysis, we have created pdacR (http://pdacR.bmi.stonybrook.edu, github.com/rmoffitt/pdacR), an open-source software package and web-tool with annotated datasets from landmark studies and an interface for user-friendly analysis in clustering, differential expression, survival, and dimensionality reduction. Using this tool, we present a multi-dataset analysis of PDAC transcriptomics that confirms the basal-like/classical model over alternatives.

## Abstract (web summary)

pdacR is an open-source R package and web-based platform that enables community exploration of pancreatic ductal adenocarcinoma (PDAC) gene expression datasets. pdacR is used to interrogate the specificities of popular PDAC molecular subtypes.

## Introduction

Over the past decade, several genomics studies of pancreatic ductal adenocarcinoma (PDAC) have contributed a wealth of molecular-level data to guide investigation of the disease. A subset of these studies have sought to define transcriptomic subtypes. Although the datasets generated from these studies are accessible as part of the public domain, bioinformatic hurdles ranging from decentralized data storage to insufficiently annotated samples complicates multi-dataset investigation, resulting in inappropriate and inconsistent application of datasets. Thus, there is an unmet need for a PDAC transcriptomic compendium that provides an easily accessible interface for analysis by the PDAC research community, while retaining the capacity for diverse analytical methods in dataset- and application-specific situations.

Multiple PDAC gene expression subtype models have been proposed toward the goal of using individualized therapeutic approaches to treat patients with this disease^1–7^. Collisson et al. first defined three prognostically-relevant PDAC-specific subtypes (classical, quasi-mesenchymal (QM), and exocrine-like) using non-negative matrix factorization consensus clustering (NMF-CC) on a merged microarray dataset comprised of laser capture microdissected PDAC tumors (*n* = 66)^2^. We later decoupled NMF and consensus clustering to create a two-subtype model (classical, with a similar gene set to Collisson’s classical type, and basal-like, similar to basal breast and bladder cancers), using microarrays from bulk primary and metastatic PDAC tumors, normal pancreas and distant site normal tissue, and PDAC cell lines (*n* = 357)^3^. Similar to Collisson et al., Bailey et al. subsequently used NMF-CC to analyze RNA sequencing (RNAseq) data from a combination of PDAC and other uncommon pancreatic cancer histological types, including acinar cell carcinomas, finding four tumor subtypes (pancreatic progenitor, squamous, aberrantly differentiated endocrine exocrine (ADEX), and immunogenic, *n* = 96)^4^. Then, in 2018, Puleo et al. proposed a five-subtype model using a consensus clustering approach on 309 formalin fixed paraffin embedded whole PDAC tumor samples (pure classical, immune classical, desmoplastic, stroma activated, and pure basal-like). However, they confirmed two-subtypes (classical and basal-like) when using only samples with purity (defined by the predicted mean variant allele frequency, or VAF) in the upper quartile of their dataset (*n* = 78)^5^.

In 2020, researchers have sought to further refine these definitions. Chan-Seng-Yue et al. utilized NMF to identify four gene signatures, subdividing basal-like and classical subtypes into “basal-like A”, “basal-like B”, “classical A”, and “classical B”^6^. Separate work by Nicolle et al. then utilized Independent Component Analysis (ICA) to generate a molecular gradient that correlates with histological differentiation and seeks to assign a continuous value to tumor samples using weighted valuations of between 4000 and 20,434 genes^7^.

Independent studies have comparatively evaluated the clinical utility of these proposed schemas. The Comprehensive Molecular Characterization of Advanced Pancreatic Ductal Adenocarcinoma for Better Treatment Selection (COMPASS) trial’s earliest results showed that the Moffitt et al. classification system provided accurate prediction of the tumors which would respond or not respond to modified FOLFIRINOX or gemcitabine/nab-paclitaxel^8^. Furthermore, Tiriac et al. showed that the basal-like/classical system could be applied to transcriptomic data from patient-derived PDAC organoids to infer patient tumors which would respond to either oxaliplatin or gemcitabine^9^, and, shortly afterward, Aguirre et al. demonstrated utility using the Moffitt subtypes to characterize metastatic PDAC biopsies for treatment guidance in a future clinical care setting^10^. In 2019, Camolotto et al. showed that knockout of the transcription factor *Hnf4a* in mice resulted in a loss of classical expression, a decrease in tissue differentiation, and worse prognosis^11^. Even more recent work by Hayashi et al. was able to show concordance between the Moffitt subtypes, morphological presentation, and clinical course^12^. In addition, O’Kane et al. used *GATA6* as a surrogate for the Moffitt basal-like/classical identification and recapitulated robust response to tumor and overall survival in the COMPASS cohort^13^. To enhance the clinical applicability of the basal-like/classical model, we previously generated a single-sample classifier termed purity independent subtyping of tumors (PurIST)^3, 14^. This classifier showed a robust ability to predict a patient’s molecular subtype and response to therapy across multiple technologies and without the need for a cohort^8^.

While a limited number of studies have begun comparisons of subtype performance in clinical care, diversity of opinions in the field and difficulty in obtaining data has led to confusion. Indeed, even recent studies show mixed strategies among investigators choosing which datasets or gene signatures to use. In particular, we highlight three common issues. First, validation is frequently only performed on one publicly available dataset. Second, there are multiple examples of inappropriate use of The Cancer Genome Atlas (TCGA) Program PDAC dataset^15–30^. The mRNA dataset includes 182 samples, but only 150 have been confirmed histologically as PDAC. The remaining 32 samples are classified as either other types of pancreatic cancer, no evidence of cancer, or adjacent normal samples. Including such samples in derivation or validation of expression signatures results in misleading results. Finally, a growing consensus surrounding the two-subtype model has led researchers to collapse other 3+ subtype models into two in an attempt to harmonize with the basal-like/classical model^31–33^. However, this approach does not address confounding factors related to purity and tissue specificity inherent in the approaches used to derive the models. Given that the two-subtype and collapsed 3+ subtype signatures are not completely redundant, attempting to interchangeably label a subset of the data as basal-like, squamous, or QM is not equivalent.

To facilitate standardized investigation of PDAC data, we have created an R package, termed pdacR, for public use. As part of this package, we have gathered and carefully annotated thirteen human PDAC datasets from the past decade for a thorough comparison of the performance of subtyping schemas in identifying only the tumor cells within PDAC tumors (Table 1). In keeping with the Findability, Accessibility, Interoperability, and Reusability (FAIR) practices promoted by the NIH, we also created a graphical user interface (GUI) for easy-to-use interrogation of these datasets in a point-and-click manner that aims to enable a wide audience to succinctly pursue hypotheses in existing PDAC transcriptomic data^34–36^. This GUI is available as web-hosted software for ease of access and use. The flexibility of pdacR, combined with its inclusion of a wide range of PDAC datasets that are focused on the most reliable, large, and commonly referenced studies, expands upon on previous work by Marzec et al. and Tan et al. to develop the Pancreatic Expression Database (PED) and Human Pancreatic Cancer Database (HPCDb), respectively, for pancreatic cancer data centralization and user interface development for genomics analysis^37–40^.

**Table 1:** Datasets in the pdacR package and those used in this analysis.

Dataset	Type	Sample description	Samples	Features	Used
Chen, 2015^52^	MA	63 fresh frozen microdissected PDAC tumor samples	63	11	no
ICGC PACA-AU, 2016^4^	MA	131 primary pancreatic tumors, 101 PDAC, 30 other	131	32	yes
ICGC PACA-AU, 2016^4^	RNAseq	82 primary pancreatic tumors, eight cell lines, and two metastatic tumors	92	32	yes
ICGC PACA-CA, 2016	RNAseq	40 cell lines, 11 xenografts, 16 metastatic tumors, and 195 primary pancreatic tumors	49	11	no
Moffitt, 2015^3^	MA	145 primary PDAC, 61 metastatic PDAC, 17 cell lines, 46 normal pancreas, 88 distant site normal tissue	357	25	yes
Moffitt, 2015^3^	RNAseq	15 primary PDAC, 37 patient-derived xenografts, three PDAC cell lines, and six cancer-associated fibroblast lines	61	7	yes
Olive, 2019^42^	RNAseq	15 triplicate-matched PDAC bulk tumor and laser capture microdissected (LCM) epithelium and stroma, 51 additional LCM epithelium-stroma pairs, and 57 additional unmatched LCM stroma	204	19	yes
Puleo, 2018^5^	MA	309 primary PDAC FFPE block tumor samples	309	19	yes
Seino, 2018^53^	MA	2 normal, 39 PDAC, 6 normal-like PDAC, and 10 genetically engineered organoids	57	11	no
TCGA-PAAD, 2017^41^	RNAseq	181 macrodissected primary tumors, with 150 whitelisted by histologic identification of neoplastic cellularity and cancer type	181	55	yes
Nones, 2013^54^	MA	103 primary pancreatic ductal adenocarcinoma samples	103	18	no
ScAtlas, 2022	scRNA	43 pseudobulked primary PDAC, 43 pseudobulked Normal pancreas	86	5	no
CPTAC3-PDA^55–57^	RNAseq	140 Primary PDAC and 39 adjacent normal pancreas	179	78	no

List of datasets included in pdacR. Dataset indicates publication where data was initially published. Type indicates method of transcriptomic measurement (MA = Microarray, scRNA = Pseudobulked scRNA). Samples indicate number of samples in the dataset. Features indicate number of associated sample metadata (e.g., Tumor grade, histology, purity, etc.). The ‘Used’ column indicates if the dataset appears in this manuscript (yes) or if it is solely provided as part of the pdacR tool (no).

## Results

### Features and organization of the pdacR package

**Fig. 1:**
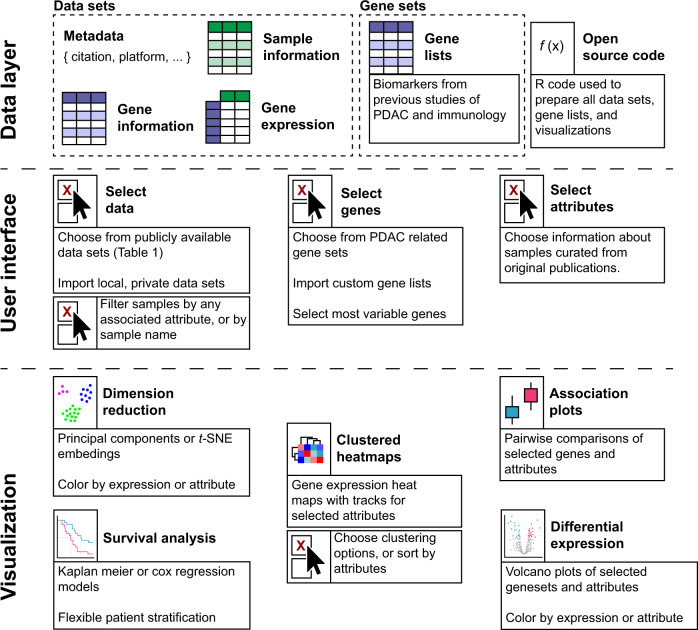
Features and organization of the pdacR package. The pdacR package contains hand-curated and annotated datasets from many of the landmark PDAC studies conducted over the past decade and others. *Data layer*: Dataset sample- and gene-based data are organized by the primary author of the dataset and the year of publication. Gene lists defined in these and other studies are compiled for ease of analysis, named by author and cell/tissue type defined by the list (e.g., “CIBERSORT Monocytes”, a gene list that specifically identifies cells of the monocyte lineage). *User interface*: A GUI that enables point-and-click is hosted online and also may be called locally by the user through the R Shiny interface. Users may select from the compiled datasets, filter samples, select genes to use in their analysis (either user-defined or built into the package), and select factors from the samples with which to visualize the data. *Visualization*: The package includes functions to facilitate data analysis and visualization, either generated for this package or from others in the literature. In the GUI, users may save images from their analysis as PNGs or PDFs for further illustration and publication.

To facilitate access and investigation of the datasets used for this paper, we compiled thirteen of the most referenced transcriptomic human PDAC datasets in an open-source R package we call pdacR. Beyond gene expression and sample information for these datasets, the package includes curated gene sets defined in earlier PDAC and immunological studies, the R code used to parse and visualize the data shown in this work, and a *shiny*-based graphical user interface (UI) for point-and-click analysis of public and user-generated data (Fig. 1).

### UI structure and data analysis

The UI is composed of four primary functional components that enable a range of investigative methods: heatmapping, dimension reduction, survival analysis, and differential expression. Users begin by choosing their dataset of choice from the list of those already curated or, on a local instance, may choose to load a user-generated dataset. User-generated datasets may be imported as R-formatted data which has been converted to a *SummarizedExperiment* container format. While the application will automatically check and convert data format from *SummarizedExperiment* to the appropriate list-style upon loading, specifics of this format and the conversion are provided on the pdacR GitHub page’s README file. Once a dataset and a gene list has been selected, many common analyses can be performed. The heatmapping component enables users to generate ordered heatmaps using user-defined gene sets and customizable clustering or sorting methods. The gene sets included are pulled directly from a variety of seminal publications in the field of PDAC transcriptomics, allowing for comparison of their performance and robustness across the available datasets. The dimension reduction component generates projections of data by either principal component analysis or t-SNE methods, with data point coloring by sample metadata or up to three gene set expression scores in an RGB color space. The survival analysis component can generate either a Kaplan Meier or Cox-based survival curve on continuous (e.g., gene expression) or categorical (e.g., tumor grade) data, and enables the user to define continuous data as categorical by toggling a quantile cutoff. The differential expression component allows users to generate volcano plots with full user freedom to define head-to-head comparisons and select which genes or gene sets to highlight.

### Comparison to other resources

Other graphical user interfaces have been developed to facilitate transcriptomics investigation in pancreatic cancer, though there are clear advantages offered in pdacR (Supplementary Table 1). Similar to the pancreatic expression database (PED) and the human pancreatic cancer database (HPCDb), pdacR combines data from several independent studies to enable hypothesis testing in different sample types from a range of datasets. PED, like pdacR, features a graphical user interface to make analyses available to users without computer programming abilities and allows users to filter their datasets based on some clinical parameters, though it is limited in other areas critical for accurate and reproducible investigation. Unlike other tools, pdacR features datasets that were parsed in a manner consistent with the recommendations of the original authors. Also, pdacR allows for extensive and flexible sample filtration prior to analysis. This is a unique feature critical to ensuring that analysis is performed on the appropriate subpopulations of datasets, while also allowing for the generation of forward-looking analyses. Importantly, the structure of pdacR as a functional R package provides investigators the ability to move their hypothesis testing from the graphical user interface to more intensive code-based analyses in an ad-hoc and specialized manner when necessary, a functionality not offered in other pancreatic cancer investigation software. Finally, pdacR uniquely combines the most frequently cited datasets from the PDAC literature with the ability for researchers to locally load their own data for ease of access to computational tools. While this package is currently limited in its ability to analyze large-scale mutation data and lacks pathways-based investigation modes, it is also, importantly, open-source to enable continuous interface development and dataset inclusion, submitted using github pull requests.

### ADEX, Exocrine, and Immunogenic subtypes are not tumor intrinsic

**Fig. 2:**
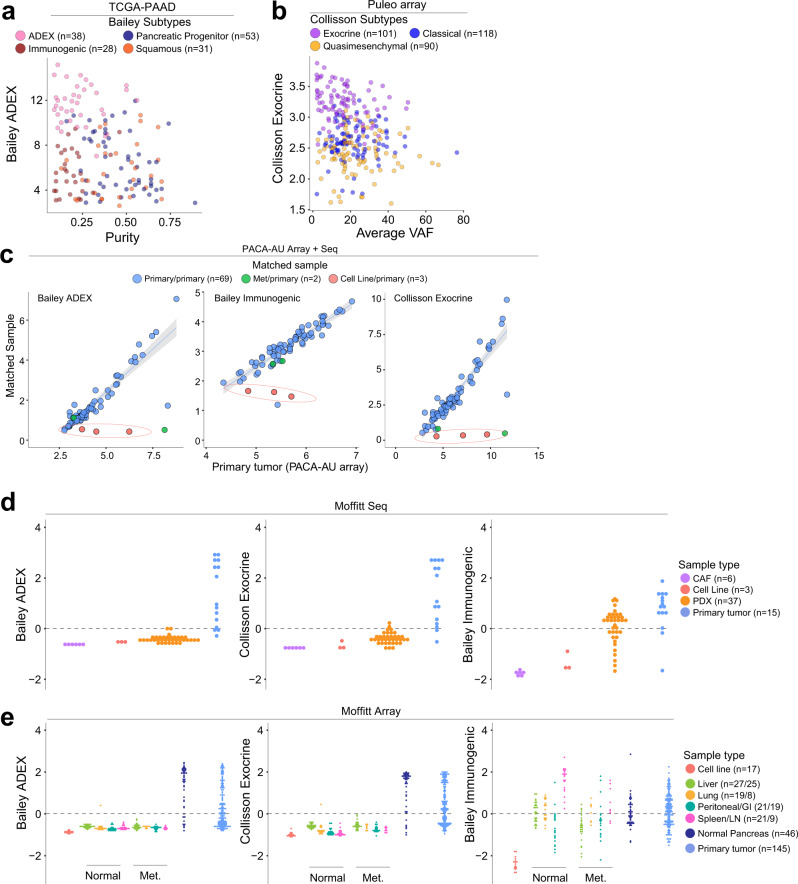
ADEX, Exocrine, and Immunogenic PDAC subtypes are not tumor-intrinsic. **a** Expression of genes in the Bailey ADEX geneset is inversely correlated with tumor sample purity, assessed by ABSOLUTE, in the TCGA dataset (Spearman rho = −0.260, *p* = 0.0014, *n* = 150). Low tumor sample purity is predictive of high ADEX geneset expression. **b** The Collisson exocrine geneset exhibits the pattern seen in the TCGA dataset (**a**), extended to the Puleo microarray (Spearman rho = −0.221, *p* = 0.0003, *n* = 309). **c** Matched samples between the PACA-AU RNAseq and PACA-AU array datasets show that the ADEX, Collisson Exocrine, and Bailey Immunogenic gene signatures are lost in cell lines and metastatic tumors derived from primary PDAC tumors expressing either high or low levels of the same gene signatures (gene expression plotted as the average of the log2 transformation of the genes which define the subtype). **d** The ADEX and Exocrine genesets are highly expressed in primary tumor samples compared to cancer-associated fibroblasts (CAFs), cell lines, and patient-derived xenografts (PDXs), though the Bailey Immunogenic geneset demonstrates appreciable expression in PDXs. **e** ADEX and Exocrine gene signatures are highly expressed in normal pancreas or primary PDAC tumors, and are comparatively absent from metastatic tumors, cell lines, and normal non-pancreas samples, whereas the Immunogenic gene signature is expressed across normal non-pancreas, metastatic, normal pancreas, and primary PDAC tumors but is lost in cell lines. Y-axes from (**d**) and (**e**) represent the mean-centered, scaled average expression for the indicated genesets.

Recent work from Rashid et al. compared the prognostic values of the Collisson, Moffitt, and Bailey subtyping schemas across clinical studies^14^. Here, we used transcriptomic data from several pancreatic cancer studies to evaluate the tumor cell specificity of these schemas. Using data from the TCGA study, we determined that high expression of genes specific to the Bailey ADEX (“ADEX”, Fig. 2a) gene signature in PDAC tumors predicts low tumor cellularity as estimated by ABSOLUTE (Spearman rho = −0.260, *p* = 0.0014) as does the Collisson exocrine gene set (Supplementary Fig. 1A). Low tumor cellularity, however, was not predictive of high ADEX gene signature expression. Using data from Puleo et al., with predicted sample average variant allele frequency (VAF) as a proxy for sample tumor cellularity, we then confirmed that the Collisson Exocrine signature is similarly predictive of low sample purity (Spearman rho = −0.221, *p* = 0.0003, Fig. 2b, association with Bailey ADEX gene set in Supplementary Fig. 2B). Importantly, these trends were not found in other gene signatures of the Collisson or Bailey subtyping schemas, suggesting that ADEX and Exocrine subtypes may be driven by expression from non-neoplastic cells.

In an analysis of paired samples (primary tumor-primary tumor, primary tumor-metastatic tumor, or primary tumor-cell line) from the PACA-AU RNAseq and PACA-AU microarray datasets, we determined that expression of the Bailey ADEX, Bailey Immunogenic, and Collisson exocrine gene signatures are lost in cell lines derived from primary PDAC tumors with a range of expression of these same signatures (Fig. 2c). Contrastingly, we found that tumor cell lines retain expression of the Collisson classical and QM, Moffitt classical and basal-like, and Bailey pancreatic progenitor and squamous subtypes (Supplementary Fig. 1). The retention of certain signatures describing both basal-like and classical populations suggests that a non-tumor cell component of the whole tumor PDACs used in this study is contributing to the expression of genes defining the ADEX, Immunogenic, and Exocrine subtypes.

**Fig. 3:**
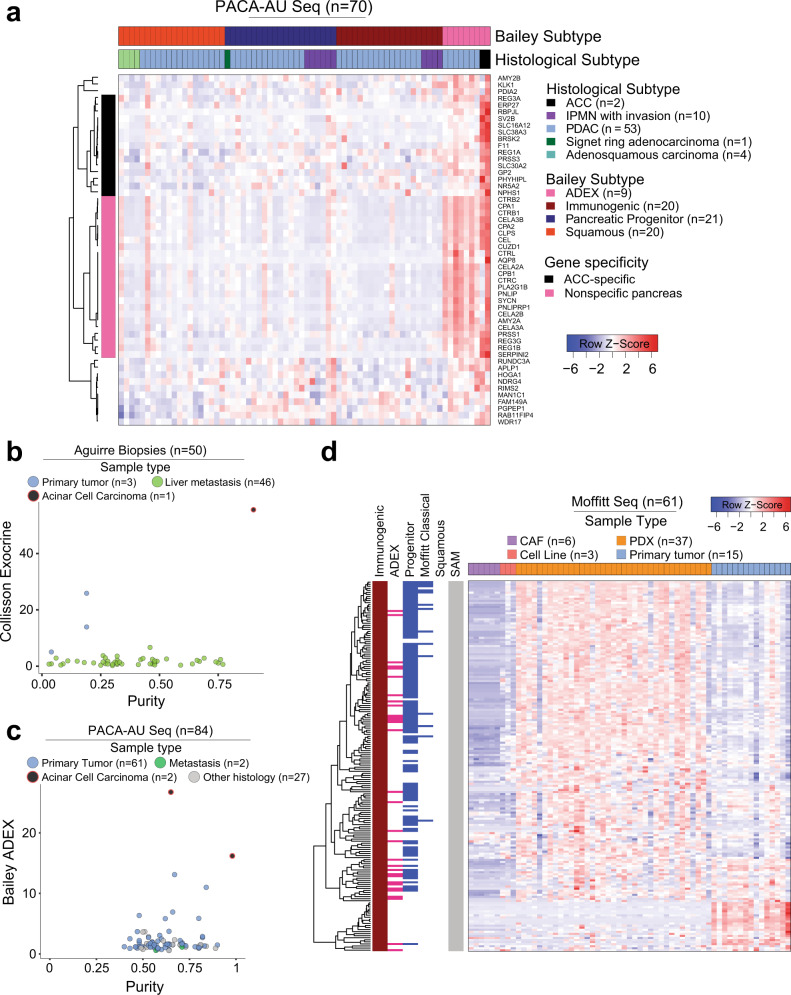
Subsets of the ADEX, Exocrine, and Immunogenic gene signatures mark non-adenocarcinoma samples. **a** The Bailey ADEX geneset is comprised of genes that specifically identify Acinar Cell Carcinomas (black), genes that appear to be more highly expressed in the ADEX subtypes yet are conserved in non-ADEX samples (light pink), and genes that are nonspecific to a known sample type (uncolored). **b**, **c** High-purity samples from core needle biopsies or primary tumor samples marked by high expression of Exocrine or ADEX expression, respectively, were histologically identified as Acinar Cell Carcinomas. **d** The subset of genes from the Immunogenic geneset that is highly expressed in PDXs is also found in the Bailey ADEX (pink), Pancreatic Progenitor (blue, left), and Moffitt classical (blue, right) signatures, while genes exclusive to the Immunogenic subtype are not expressed in PDXs.

To further assess the specificity of existing subtype gene signatures, we used RNAseq and microarray data from Moffitt et al., which includes primary PDACs, patient-derived xenografts, PDAC cell lines, cancer-associated fibroblasts (CAFs), and normal and metastatic PDAC tissue from liver, lung, peritoneum/stomach/intestine, and spleen/lymph node. We found that the ADEX, Exocrine, and Immunogenic gene sets are highly expressed in primary PDACs compared to either PDAC cell lines or subcutaneously implanted patient-derived xenografts (PDXs). Notably, primary PDACs also demonstrate increased expression of these gene sets compared to CAF cell lines (*p* < 0.0001), suggesting that the non-tumor component driving ADEX, Exocrine, and Immunogenic gene expression is unlikely dominated by CAFs (Fig. 2d). Moffitt basal-like and classical subtypes were similarly resistant to confounding by CAF gene expression (Supplementary Fig. 1B). Similarly, this trend is maintained across the Collisson classical/QM subtypes and the Bailey progenitor/squamous subtypes, which are highly concordant (~60% overlap) with the Moffitt classical/basal-like subtypes, respectively (Supplementary Fig. 1b, c). Importantly, we found that the ADEX/Exocrine signatures are enriched in normal pancreata compared to PDACs (*p* < 0.0001), suggesting a pancreas cell intrinsic ADEX/Exocrine gene expression origin (Fig. 2e). The Bailey Immunogenic subtype, however, was enriched in normal pancreas, metastatic tumor, and primary PDACs compared to cell lines (*p* < 0.0001), suggesting that a non-tumor cell component drives expression of this gene set across several tissue types (Fig. 2e). Contrastingly, though Moffitt basal-like and classical subtypes demonstrate varied representation across normal pancreas, primary pancreatic tumor, and metastatic tumor, expression of these subtypes is retained in PDAC cell lines, with similar expression patterns to the analogous Collisson and Bailey subtypes (Supplementary Fig. 1C). It is important to note that there exist pancreatic tumors of high tumor cell purity that also show high expression of the ADEX and Exocrine gene sets. Histologic characterization of these tumors, however, reliably identifies these tumors as acinar cell carcinomas (ACCs), not PDAC (Fig. 3b, c, Supplementary Fig. 2c).

### Subsets of the ADEX, Exocrine, and Immunogenic gene signatures mark non-adenocarcinoma samples

After identifying that the ADEX, Exocrine, and Immunogenic gene signatures are not tumor cell specific, we hypothesized that expression of these gene sets might be confounded by subsets of genes which are not PDAC-specific. To determine if this were the case, we performed gene-based consensus clustering of the PACA-AU RNAseq and Moffitt RNAseq datasets. We found that the Bailey ADEX gene set is comprised of primarily three subsets of genes: those that are generally nonspecific, those that are pancreas nonspecific, and those that are ACC-specific. This could explain why ADEX signatures are enriched in ACC samples, and provides justification for the non-tumor-specific nature of the signature (Fig. 3a).

A similar analysis of the Bailey Immunogenic gene set demonstrated 26% overlap with the pancreatic progenitor and 9% of the ADEX gene sets, suggesting a lack of specificity in the signature. Furthermore, we identified that 24 of the 180 immunogenic genes used in this analysis are highly expressed in primary PDACs but not PDXs, suggesting that these genes likely confer non-tumor specificity to the signature (Fig. 3d). In order to reliably investigate the role of tumor or stromal expression patterns on response to therapy and survival, the signals must be clearly separable by tissue compartment.

### Large gene sets are confounded by expression in adjacent normal tissues

Gene sets become increasingly prone to noise and off-target effects as they increase in length and include more biologically multi-functional transcripts. For example, normal pancreas expresses even the neoplastic Collisson and Bailey gene sets (Supplementary Fig. 1D, E). The lists extracted from Chan-Seng-Yue et al. also show aberrant expression in a range of normal tissues as well as susceptibility to confounding by CAFs specifically in the Basal-like A list (Supplementary Fig. 3B, C). As an addition to categorical approaches, Nicolle et al. recently developed a Pancreatic Molecular Gradient (PAMG) to assign a continuous score that correlates with differentiation status and survival^77^. The signature, derived from primary tumors and PDXs, incorporates up to 20,164 genes into scoring. As seen in other signatures, PAMG application results in a varying range of scores across purities and tissue types (Supplementary Figs. 3, 4). The basal-like/classical signatures were purposefully derived with this problem in mind (Moffitt 2015). As such, they are robust against both metastatic location (Supplementary Fig. 1C) and neoplastic purity (Supplementary Fig. 4)^41^. Thus, longer signatures result in more complex and potentially less useful tools than the basal-like/classical or PurIST models. The above data together suggest that in the context of unpurified samples, the basal-like/classical signatures or PurIST classifier should be used in favor of seemingly similar models.

## Discussion

Several studies have proposed PDAC transcriptomic subtypes. Though efforts have been made to explain reasons for the differences between these proposals, widespread uncertainty is evident in the PDAC literature regarding which subtyping schemas are most relevant for prospective studies of tumor cells^31^. Here we used transcriptomic data from several landmark PDAC studies to understand which subtypes are the most tumor-cell specific. We show that the basal-like/classical and PurIST two-subtype schemas specifically are the most likely to identify tumor cell components of PDAC tumors across studies, reinforcing and expanding upon early suggestions by TCGA and Maurer et al.^41, 42^.

One goal of describing molecular subtypes is to predict patient outcomes and response to therapy. We previously have shown that the logistic regression classifier PurIST remains robust across multiple datasets at predicting patient outcomes^14^. A benefit of our classifier is the lack of variation between datasets, cohorts, and data types. By utilizing pre-defined top-scoring pairs of genes, PurIST can be applied to a single patient and generate a prediction without the need for a cohort. This reduces uncertainty and promotes reliability regardless of available technology or dataset size. Conversely, continuous scores such as PAMG introduce uncertainty at the clinical level due to the requirement for normalization. This makes the score, and therefore the prediction, cohort dependent.

Beyond needing clarity regarding the clinical application and tumor-intrinsic quality of transcriptomic subtypes, the PDAC research field currently lacks data centralization. Furthermore, others have indicated that data that is readily accessible through online repositories has, in some cases, been either poorly formatted or poorly annotated for subsequent analysis^43, 44^. In certain instances, this may lead to misguided or misinformed conclusions in well-meaning investigations^15–30^. Clearly, these challenges pose a major hurdle in the development of novel hypotheses, particularly for those without first-hand familiarity with how the data were generated. To remedy this, we developed an R package termed pdacR that houses carefully organized and annotated public PDAC transcriptomics data for ease of access and heightened transparency. pdacR includes a point-and-click user interface to facilitate investigation of these datasets by researchers without formal training in computational statistics in an effort to democratize data access. We have shown here that the pdacR user interface provides the user with a wide variety of data visualization methods that are highly customizable yet user-friendly. Importantly, pdacR improves on existing pancreatic cancer user interfaces, namely by retaining its flexibility as a user interface and R package, with full customization by the experienced statistician. We expect that this tool will become widely adopted in the PDAC community, especially as RNA quantification methods become more widely used to study this disease.

We have presented data to suggest that the basal-like/classical subtyping schema is both the most clinically reliable model and the most appropriate in describing only the tumor cells within a non-purified PDAC tumor. This data and others are now compiled in a public R package, pdacR, for open use by the research community. While this work has helped further our understanding of PDAC subtypes, future studies on these subtypes in the context of patient outcomes and response to therapy are necessary to apply our understanding to clinically meaningful intervention strategies. We hope this tool (http://pdacR.bmi.stonybrook.edu) and the associated code and data (github.com/rmoffitt/pdacR) will be used by other researchers to facilitate robust external validation of future analyses.

Recent attempts to improve our understanding of PDAC have emphasized the utility of single-cell RNA sequencing (scRNA). Given that the datasets provided here are all bulk analyses, we are limited in our ability to fully separate cellular populations. We partially address this shortcoming using datasets derived from a multitude of pure cell type sources or those enhanced by LCM. While scRNA sequencing is expanding in its utilization, it remains prohibitively expensive for inclusion in the realm of clinical characterization and application. While incorporation of new scRNA datasets would improve this tool from a purely investigative standpoint, the computational burden and memory required are beyond the scope of a responsive GUI. We have, in parallel, made available a parsed single-cell atlas (https://github.com/rmoffitt/scOh). This atlas does not include a web interface, but covers many substantial hurdles in scRNA analysis, including parsing, integration, and normalization. Another limitation inherent to this sort of data collection is the inability to robustly merge datasets. Preliminary versions of this tool did have the option for data aggregation. However, in addition to being too computationally intensive for a point-and-click interface, we found that aggregation introduced more problems than it solved. If we cannot assume that datasets come from comparable distributions and cell type compositions (e.g., all samples in both datasets are primary tumors), then we necessarily confound our analysis. In addition, our datasets come from a variety of platforms, including bulk RNA sequencing and microarrays, adding another layer of complexity to aggregation and batch correction. Believing that providing the option of poorly-merged data would be more harmful than helpful, we ultimately decided was beyond the scope of this project. It is our belief that the rapidity with which a researcher could perform serialized analysis on different datasets overcomes much of the limitation of not being able to merge the data.

## Methods

### Data acquisition and processing

All datasets used in this study were obtained from public repositories, indicated in Table 1. All data were previously publicly available and were collected under the approval of the respective IRBs at the time of collection and publication of the respective study. Expression matrices were unmodified compared to the original publication, without re-alignment of RNAseq data, or re-normalization of array data.

### Gene expression analysis

Gene expression analyses were performed using log2-scaled expression matrices. Heatmaps were generated using the “heatmap.3” function in R. Gene set scores were called as the mean expression value of the genes in gene set. For correlation between the PACA-AU RNAseq and Micro Array, these gene set scores were compared directly. For evaluation of expression across different tissue types in Moffitt Array and Moffitt Seq, these scores were mean-centered and scaled.

### Clustering

For clustering and subtype calls (when lacking in original publication), datasets were trimmed down to include only PDAC samples and only the genes present in the relevant gene sets. We then applied the ConsensusClusterPlus function with kmeans clustering and euclidean distance to call the appropriate number of cluster (k = number of gene sets).

### Statistics and reproducibility

To account for the potential disproportionate impact of outliers on correlations, correlation values were calculated using Spearman Rho. Difference in expression of gene sets by cell types was calculated using a non-parametric Wilcoxon Rank-Sum test. Reproducibility is one of our main motivators for this publication. To that end, we have provided a web application wherein all the analyses can be replicated with a series of clicks. We have also provided all our analysis code, along with parsed and ready-to-analyze datasets, in our github^45^.

### R package and UI design

The pdacR UI was designed using the *shiny* R package. Datasets were chosen for inclusion in the pdacR package based on public availability or investigator permission, as well as importance in the field as determined by the authors. Differential expression analysis on RNAseq or Array experiments is performed using the DESeq2^46^ or limma^47^ packages respectively. The logFC is calculated by dividing the mean of cohort A by the mean of cohort B for each gene. Survival analysis is performed using the survival and survminer packages in R. Censor and survival data are obtained from the initial publications, and are a combination of overall and disease-specific survivals. User-generated data may be converted from a *SummarizedExperiment* container style to a UI-compatible format using our ‘./R/Convert_GUI_data.R’ wrapper function.

### Reporting summary

Further information on research design is available in the Nature Portfolio Reporting Summary linked to this article.

## Supplementary information

Supplementary Information

Reporting Summary

## Data Availability

All datasets used in this study are compiled in our pdacR package, which is freely available to the public at github.com/rmoffitt/pdacR/data. All datasets have been pulled from publicly accessible databases, and their accessions are contained within the metadata of each R object. Accession numbers are also listed here: Chen - GSE57495; CPTAC - phs001287; Nones - GSE50827; Bailey (PACA-AU) - EGAS00001000154; PACA-CA - icgc.org; Moffitt Array - GSE71729; Moffitt Seq - PMID:26343385 (Supplement); Olive - GSE93326; Puleo - E-MTAB-6134; Seino - GSE107610; TCGA - phs000178. Within R, these data can be pulled from their original sources using packages such as TCGAbiolinks^48–50^ or GEOquery^51^, but we recommend using our pre-parsed formats.

## Code availability

All parsing and analytical code used in this study are compiled in our pdacR package, which is available to the public at github.com/rmoffitt/pdacR. Figures were generated using the .rmd files in inst/analysis, while the entire app framework is located at inst/shiny/app.R. The Github version at time of manuscript preparation can be identified by SHA: 3a3f5683059b943cb3ef2d762ab3cddccd572bb0^45^.
