# Supplementary material for: Open-source curation of a pancreatic ductal adenocarcinoma gene expression analysis platform (pdacR) supports a two-subtype model
Source: Commun Biol. 2023 Feb 10;6:163. doi: 10.1038/s42003-023-04461-6 (PMC9918476; doi:10.1038/s42003-023-04461-6)
Supplement: Supplementary file 1 — Supplementary Information [file 42003_2023_4461_MOESM1_ESM.pdf]

SUPPLEMENTARY FIGURES

Supplementary Figure 1

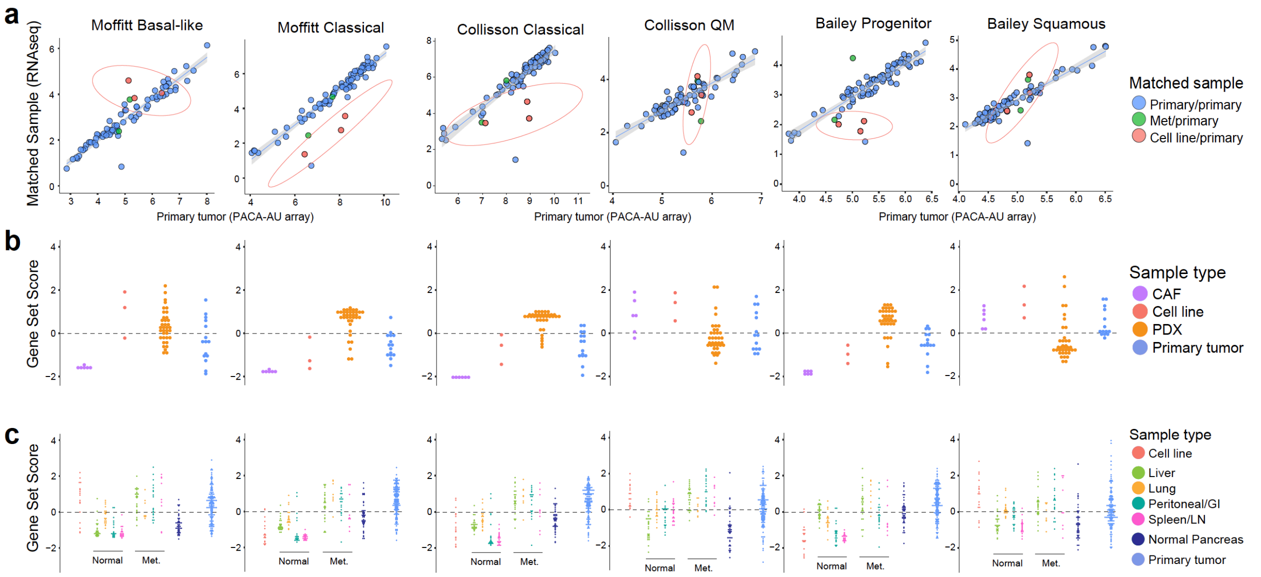

Supplementary Figure 1:
Moffitt basal-like and classical subtypes resist non-tumor confounding effects
a, Signatures that are not confounded by purity are retained in cell lines and metastatic tumors derived from primary PDAC tumors expressing either high or low levels of the same gene signatures (gene expression plotted as the mean of the log2 transformation of the genes which define the subtype for both axes). **b**, Collisson QM and Bailey Squamous are similarly expressed in CAFs when compared to pancreatic cancer cell lines, PDXs, and primary tumors. Moffitt signatures are both expressed to lesser amounts in CAF when compared to other cell types. **c**, basal-like and classical genesets are expressed in pancreatic cancer cell lines in addition to metastatic and primary pancreatic tumors, with limited expression in normal tissues. Y-axes from (**b**) and (**c**) represent the mean-centered, scaled average expression for the indicated genesets.

Supplementary Figure 2

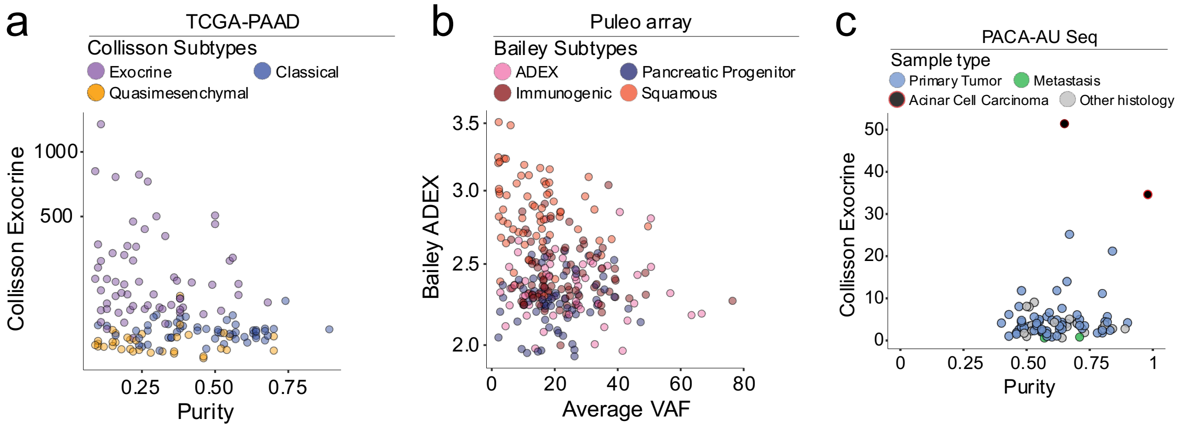

Supplementary Figure 2:
Exocrine and ADEX subtypes are confounded by tumor purity and non-adenocarcinoma samples
a, Low tumor purity is predictive of high Collisson exocrine geneset expression, and Collisson exocrine geneset expression is negatively correlated with tumor purity (Spearman rho = -0.22097 p = .0068). **b**, Expression of the Bailey ADEX geneset in primary PDACs is negatively correlated (Spearman rho = -0.19677, p =.0012 with VAF, a proxy for tumor purity. **c**, The Collisson exocrine geneset is highly confounded by Acinar Cell Carcinoma (ACC) gene expression, similar to the confounding effect seen in ACCs with the Bailey ADEX geneset.

Supplementary Figure 3

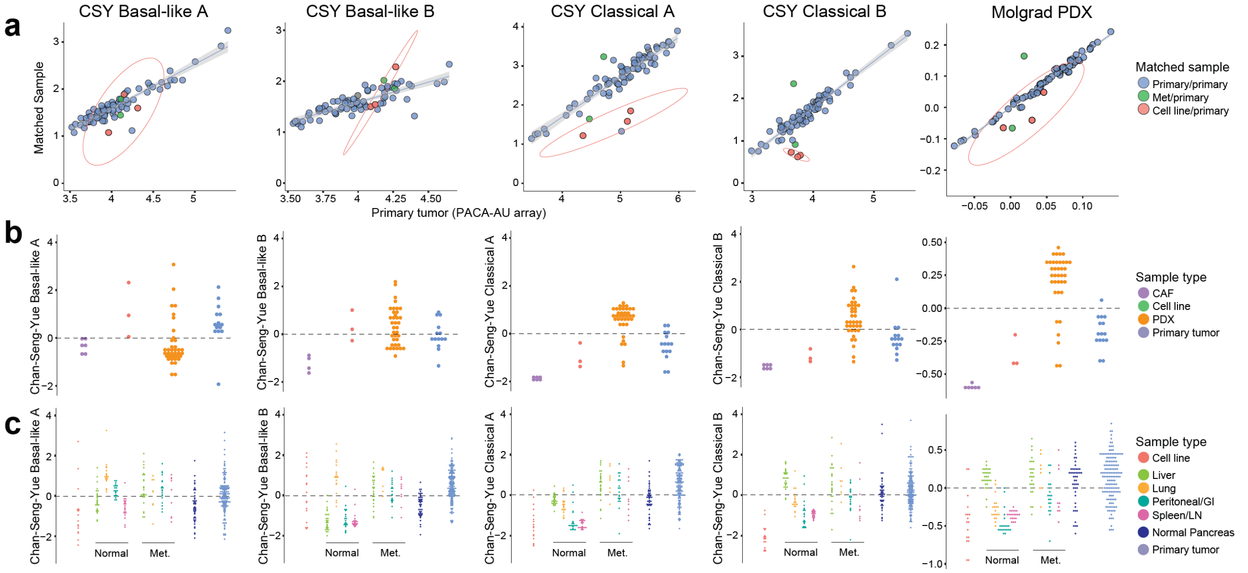

Supplementary Figure 3:

Chan-Seng-Yue basal-like and classical subtype subdivisions are somewhat resistant to non-tumor confounding effects

a, Analysis of matched samples between the PACA-AU RNAseq and PACA-AU array datasets demonstrates that the Chan-Seng-Yue basal-like A and B and classical A and B signatures, as well as PAMG expression, are retained in cell lines and metastatic tumors derived from primary PDAC tumors expressing either high or low levels of the same gene signatures (gene expression plotted as the average of the log2 transformation of the genes which define the subtype). **b**, The Chan-Seng-Yue basal-like and classical signatures are generally resistant to confounding effects from CAFs and are all found in pancreatic cancer cell lines, PDXs, and primary tumors. The Chan-Seng-Yue basal-like A signature, however, demonstrates appreciable expression in CAFs and **(c)** is retained in normal lung and peritoneal/GI. Y-axes represent the mean-centered, scaled average expression for the indicated genesets.

Supplementary Figure 4

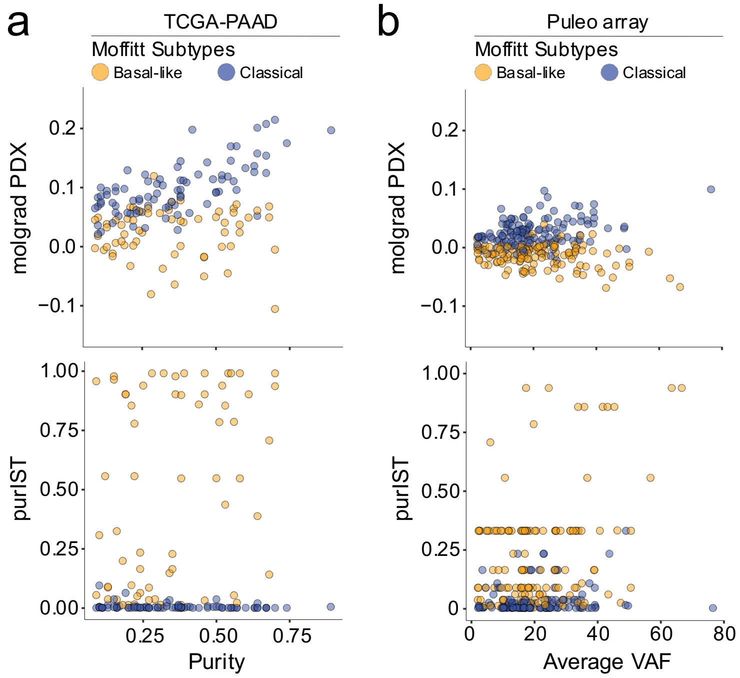

Supplementary Figure 4:
Molgrad score distributions are dataset-dependent
a, The Molgrad PDX (top) and PurIST (bottom) subtyping schemas across tumor sample purity as assessed by ABSOLUTE. Data are plotted as unscaled outputs from the respective methods. **b**, Same as **a**, but using sample average VAF as a proxy for tumor purity, from the Puleo array dataset. 'basal-like' in the PurIST method are described as samples with scores >= 0.5, while Molgrad PDX method associates negative values with basal-like characteristics.

Supplementary Table 1

Function	pdacR	HPCDb[1]	PED[2-4]
GUI	X		X
Choice of Statistical Method	X		
Flexible for analysis complexity	X		
RNAseq and Microarray	X		X
Single Cell RNAseq capable	X		
Human and Mouse capable	X		
Publication-quality visualization	X		X
No Programming required	X	X	X
PubMed dataset querying			X
Dataset curation/annotation accurate to publication	X		
Non-transcriptomic genomics data			X

Supplementary Table 1: Comparison of pdacR with other PDAC genetics tools.
An X in a row indicates that the tool in question has that feature.

References

1 Tan, Y. *et al.* HPCDb: an integrated database of pancreatic cancer. *bioRxiv*, 169771 (2017). https://doi.org:10.1101/169771

2 Chelala, C. *et al.* Pancreatic Expression database: a generic model for the organization, integration and mining of complex cancer datasets. *BMC Genomics* **8**, 439 (2007). https://doi.org:10.1186/1471-2164-8-439

3 Dayem Ullah, A. Z. *et al.* The pancreatic expression database: recent extensions and updates. *Nucleic Acids Res* **42**, D944-949 (2014). https://doi.org:10.1093/nar/gkt959

4 Marzec, J. *et al.* The Pancreatic Expression Database: 2018 update. *Nucleic Acids Res* **46**, D1107-d1110 (2018). https://doi.org:10.1093/nar/gkx955
