# Supplementary material for: Open-source curation of a pancreatic ductal adenocarcinoma gene expression analysis platform (pdacR) supports a two-subtype model
Source: Commun Biol. 2023 Feb 10;6:163. doi: 10.1038/s42003-023-04461-6 (PMC9918476; doi:10.1038/s42003-023-04461-6)
Supplement: Supplementary file 2 — Reporting Summary [file 42003_2023_4461_MOESM2_ESM.pdf]

nature portfolio

Corresponding author(s): Richard Moffitt

Last updated by author(s): Nov 9, 2022

Reporting Summary

Nature Portfolio wishes to improve the reproducibility of the work that we publish. This form provides structure for consistency and transparency in reporting. For further information on Nature Portfolio policies, see our Editorial Policies and the Editorial Policy Checklist.

Statistics

For all statistical analyses, confirm that the following items are present in the figure legend, table legend, main text, or Methods section.

n/a	Confirmed	
☐	☒	The exact sample size (*n*) for each experimental group/condition, given as a discrete number and unit of measurement
☐	☒	A statement on whether measurements were taken from distinct samples or whether the same sample was measured repeatedly
☐	☒	The statistical test(s) used AND whether they are one- or two-sided<br>*Only common tests should be described solely by name; describe more complex techniques in the Methods section.*
☒	☐	A description of all covariates tested
☒	☐	A description of any assumptions or corrections, such as tests of normality and adjustment for multiple comparisons
☐	☒	A full description of the statistical parameters including central tendency (e.g. means) or other basic estimates (e.g. regression coefficient) AND variation (e.g. standard deviation) or associated estimates of uncertainty (e.g. confidence intervals)
☐	☒	For null hypothesis testing, the test statistic (e.g. *F*, *t*, *r*) with confidence intervals, effect sizes, degrees of freedom and *P* value noted<br>*Give P values as exact values whenever suitable.*
☒	☐	For Bayesian analysis, information on the choice of priors and Markov chain Monte Carlo settings
☒	☐	For hierarchical and complex designs, identification of the appropriate level for tests and full reporting of outcomes
☐	☒	Estimates of effect sizes (e.g. Cohen's *d*, Pearson's *r*), indicating how they were calculated

Our web collection on statistics for biologists contains articles on many of the points above.

Software and code

Policy information about availability of computer code

Data collection

All data collection and parsing scripts are made available at github.com/rmoffitt/pdacr/R/

Raw data accession codes are provided as part of each dataset object's metadata, and accession of this information is detailed in the readme at github.com/rmoffitt/pdacr/readme. In addition, raw accession is displayed when datasets are loaded into the webtool

Data analysis

Data analysis was performed using the following packages and scripts.
- Heatmapping was performed using heatmap.3, getSideColors, hclust, and ConsensusClusterPlus
- Cartesian analysis was performed using rtsne and pca
- Survival analysis was performed using survival and survminer
- Differential Expression analysis was performed using DESeq2 and limma

Please note, all helper functions are made available at github.com/rmoffitt/pdacr/R, while all analysis documents are found at github.com/rmoffitt/pdacr/inst/analysis. Analytical code is all integrated into the webtool found at http://pdacr.bmi.stonybrook.edu, and the raw code is made available at github.com/rmoffitt/pdacr/inst/shiny/app.R

For manuscripts utilizing custom algorithms or software that are central to the research but not yet described in published literature, software must be made available to editors and reviewers. We strongly encourage code deposition in a community repository (e.g. GitHub). See the Nature Portfolio guidelines for submitting code & software for further information.

Data

Policy information about availability of data

All manuscripts must include a data availability statement. This statement should provide the following information, where applicable:

- Accession codes, unique identifiers, or web links for publicly available datasets
- A description of any restrictions on data availability
- For clinical datasets or third party data, please ensure that the statement adheres to our policy

All datasets used in this study are compiled in our pdacR package, which is available to the public at github.com/rmoffitt/pdacR.

Human research participants

Policy information about studies involving human research participants and Sex and Gender in Research.

Reporting on sex and gender	This study does not involve human participants
Population characteristics	This study does not involve human participants
Recruitment	This study does not involve human participants
Ethics oversight	This study does not involve human participants

Note that full information on the approval of the study protocol must also be provided in the manuscript.

Field-specific reporting

Please select the one below that is the best fit for your research. If you are not sure, read the appropriate sections before making your selection.

☒ Life sciences ☐ Behavioural & social sciences ☐ Ecological, evolutionary & environmental sciences

For a reference copy of the document with all sections, see nature.com/documents/nr-reporting-summary-flat.pdf

Life sciences study design

All studies must disclose on these points even when the disclosure is negative.

Sample size	We compiled 13 datasets totaling 1,872 samples. These publicly available datasets represent seminal PDAC datasets that are often cited along with the derivation of molecular signatures and clinical findings.
Data exclusions	No data were excluded from overall analyses.
Replication	One of our main rationales for compiling all of these datasets is to ensure replicability of findings. Findings were demonstrated in multiple datasets when sufficient clinical and molecular information was provided.
Randomization	As a non-experimental study, no interventional experiments were designed and therefore no randomization was relevant.
Blinding	Investigators were not blinded during analysis

Reporting for specific materials, systems and methods

We require information from authors about some types of materials, experimental systems and methods used in many studies. Here, indicate whether each material, system or method listed is relevant to your study. If you are not sure if a list item applies to your research, read the appropriate section before selecting a response.

Materials & experimental systems

n/a	Involved in the study
☒	☐ Antibodies
☒	☐ Eukaryotic cell lines
☒	☐ Palaeontology and archaeology
☒	☐ Animals and other organisms
☒	☐ Clinical data
☒	☐ Dual use research of concern

Methods

n/a	Involved in the study
☒	☐ ChIP-seq
☒	☐ Flow cytometry
☒	☐ MRI-based neuroimaging
